# The *WUSCHELa* (*PtoWUSa*) is Involved in Developmental Plasticity of Adventitious Root in Poplar

**DOI:** 10.3390/genes11020176

**Published:** 2020-02-06

**Authors:** Jianbo Li, Huixia Jia, Pei Sun, Jin Zhang, Yongxiu Xia, Jianjun Hu, Lijuan Wang, Mengzhu Lu

**Affiliations:** 1 Experimental Center of Forestry in North China, Chinese Academy of Forestry, Beijing 102300, China; Lijb2017@caf.ac.cn (J.L.); yxxia@caf.ac.cn (Y.X.); 2 State Key Laboratory of Tree Genetics and Breeding, Chinese Academy of Forestry, Beijing 100091, China; Jiahx@caf.ac.cn (H.J.); Sunpei@caf.ac.cn (P.S.); Hujj@caf.ac.cn (J.H.); 3 Research Institute of Forestry, Chinese Academy of Forestry, Beijing 100091, China; 4 Biosciences Division, Oak Ridge National Laboratory, Oak Ridge, TN 37831, USA; zhangj1@ornl.gov; 5 College of Forestry and Biotechnology, Zhejiang Agriculture & Forestry University, Hangzhou 311300, China

**Keywords:** adventitious roots, auxin, poplar, *PtoWUSa*, transgenic plant

## Abstract

*WUSCHEL-RELATED HOMEOBOX* (*WOX*) transcription factors play critical roles in cell fate determination during plant development. As the founding member of the *WOX* family, *WUSCHEL* (*WUS*) is characterized for its role in maintaining stem cell in meristem. In this study, we investigated the function of *Populus tomentosa WUSCHELa* (*PtoWUSa*) in adventitious roots (ARs) in poplar. Expression profile analysis showed that *PtoWUSa* was not only expressed in shoot apical meristem and stem, but also expressed in ARs. Ectopic expression of *PtoWUSa* in *Arabidopsis* resulted in shortened primary root, as well as agravitropism and multiple branches. Overexpression of *PtoWUSa* in poplar increased the number of ARs but decreased their length. Moreover, the AR tip and lateral root tip became larger and swollen. In addition, the expression of auxin transporter genes *PIN-FORMED* were downregulated in ARs of transgenic plant. Taken together, these results suggest that *PtoWUSa* could be involved in AR development in poplar through regulating the polar auxin transport in ARs.

## 1. Introduction

Cutting is a main method of vegetative propagation for woody plants. An essential step of cutting depends on the formation and development of adventitious roots (ARs). Therefore, dissecting the molecular mechanisms of the development of ARs and exploring the function of genes regulating adventitious rooting will be beneficial for improving propagation efficiency. ARs are always generated from the stem-, hypocotyl- or leaf-derived cells. The development of ARs is regulated by multiple endogenous and environmental factors. Among them, phytohormone auxin plays a key role [1]. Auxin is mainly synthesized in young aerial parts and flows down to the root tip columella in which an auxin maximum is formed, and then transports to other parts of the root through root epidermal cells [2]. During this process, the formed auxin concentration gradients regulate the root growth and plasticity. The auxin transport from cell-to-cell is mediated by auxin transport carriers, such as AUX1-like influx carrier, PIN-FORMED efflux carrier (PIN) and P-glycoprotein auxin carrier (PGP) [3]. The PIN is characterized by its polar plasma membrane localization that regulates the direction of auxin flow [4]. PGPs can improve the stability of PINs in plasma membrane and enhance cellular auxin transport activity [5]. PIN, PGP efflux transporters, and AUX1 influx transporters determine the directionality of intercellular auxin flow [6]. One of the prominent growth responses regulated by auxin polar transport is root tropism, such as the mutants of *PIN2*, *PIN3*, *PIN5*, *PIN7,* and *AUX1* with root gravitropism [7,8,9,10,11].

The *WUSCHEL*-*related homeobox* (*WOX*) gene family is a plant-specific transcription factor and belongs to the homeodomain superfamily [12]. The function of *WOX* genes in *Arabidopsis thaliana* have been well characterized and show that *WOXs* play critical roles in stem cell maintenance in shoot and root apical meristems (SAM and RAM), root development, et al. [13,14,15]. *AtWOX1*/*AtWOX3* regulates lateral axis-dependent development of lateral organs [16]. *AtWOX2*, *AtWOX8* and *AtWOX9* are required for apical-basal axis formation of pre-embryo, and *AtWOX9* also plays a role in maintaining the cell division in early embryogenesis [17,18]. *AtWOX4* is expressed in vascular and regulates the vascular stem cell proliferation, and it acts redundantly with *AtWOX14* in the maintenance of vascular meristem organization during secondary growth [19,20]. *AtWOX5* is specifically expressed in the cells of quiescent center and functions in organizing stem cells in RAM [21,22]. *AtWOX7* regulates the lateral root (LR) development [23]. *AtWOX11*/*12* are involved in AR de novo organogenesis by activating the expression of *AtWOX5* and *AtWOX7* [24,25].

As the founding member of *WOX* family, *WUSCHEL* (*WUS*) is originally identified in *Arabidopsis* [26]. *AtWUS* serves as a central regulator in the maintenance of stem cell identity in the central zone of SAM and floral meristems [26,27]. *AtWUS* expression is restricted in organizing center of SAM, and AtWUS protein can move to the stem cell region to promote the expression of *CLAVATA 3* (*CLV*3), then the activated *CLV3* signaling pathway inhibits the expression of *WUS*, resulting in maintaining stem cell homeostasis in SAM [28,29,30]. At the reproductive phase, the *WUS*-*AGAMOUS* feedback loop controls the flower meristem (FM) identity [31]. In *atwus* mutant, plants develop in stop-and-go mode, and defective SAM and FM are initiated repetitively, leading to many disorganized bunches of leaves along the plant [26]. In *Oryza sativa*, *OsWUS* is expressed in young leaf primordia, axillary meristems (AMs) and transient expressed in rice SAM [32,33,34]. *OsWUS* is required for initiation of the AMs, but not for maintenance of the SAM [34]. The mutants of *OsWUS*, *tab1*-*1* [24], *moc3*-*1* [33], and *srt* [35] exhibit various degree defects in tillering and flower development. Overexpression of cotton (*Gossypium hirsutum*) *GhWUS* can induce somatic embryo and shoot formation [36]. Despite the function of *WUS* in developmental processes, which has been studied in herbaceous plant, little is known about its function in woody plants.

As a model woody species, *Populus* is characteristic with rapid growth and high production of plant biomass. In our previous study, 18 *WOX* genes were identified in *Populus tomentosa*, and 2 *P. tomentosa WOXs* (*PtoWUSa* and *PtoWUSb*) were identified as the orthologous genes of *Arabidopsis AtWUS* [37]. In this study, we characterized the function of *PtoWUSa* in AR development. Expression profile analysis showed that *PtoWUSa* was not only expressed in SAM and stem, but also expressed in ARs. Transgenic results displayed that overexpression of *PtoWUSa* affected the root morphogenesis by down-regulation of *PIN* expressions.

## 2. Materials and Methods

### 2.1. Plant Materials and Growth Conditions

*A. thaliana* (ecotype Columbia-0) was grown at 20–22 °C under 16 h/8 h light/dark photoperiod in 1/2 Murashige-Skoog (MS) medium or soil. Hybrid poplar (*P*. *alba* × *P*. *glandulosa*) 84K was performed tissue culture to obtain poplar clonal plants for transgenic acceptors and was grown at 23–25 °C under 16 h/8 h light/dark photoperiod.

### 2.2. Domain, Conserved Motifs and Phylogenetic Sequence Analysis

The WUS amino acid sequences of different species were obtained from website (http://www.phytozome.com, https://www.ncbi.nlm.nih.gov/genbank/). The multiple sequences alignment of WUS was generated using DNAMAN 7.0 (Lynnon Corporation, San Ramon, USA). The GenBank accession numbers of the WUS from different species were: AtWUS (*A. thaliana*, AT2G17950), OsWUS (*O. sativa*, BAE48303.1), PtoWUSa (*P. tomentosa*, AHL29308.1), PtoWUSb (*P. tomentosa*, ACO55494.1), PtrWUSa (*P. trichocarpa*, Potri.005G114700), PtrWUSb (*P. trichocarpa*, Potri.007G01210), SsuWUS (*Salix suchowensis*, SapurV1A.0487s0060.1), SLWUS (*Solanum lycopersicum*, NP_001234015.2), VvWUS (*Vitis vinifera*, XP_002266323.1). Clustal X2 was used for sequence alignment, and MEGA v6.0 was used to construct phylogenetic tree with neighbor-joining (NJ) method with 1,000 bootstrap replicates.

### 2.3. RNA Extraction and qRT-PCR Assays

Total RNAs were extracted from plant samples using the RNeasy Plant Mini Kit (Qiagen, Hilden, Germany). The quality and concentration of RNAs were detected by agarose gel electrophoresis and NanoDrop 8000 (Thermo, Wilmington, USA). The first stand cDNA was acquired using SuperScript III first-strand synthesis system (Life technologies, Carlsbadn, USA) with 1 μg mRNA. Quantitative real-time PCR (qRT-PCR) was performed on the Roche LightCycler 480 (Roche Applied Science, Penzberg, Germany) following manufacturer’s instructions. *Actin7* and *PtActin* were chose as reference genes. The 2^−ΔΔCT^ method was used to calculate the relative expression level [38]. Three biological replicates and four technical replicates of each sample were performed for qRT-PCR analysis. The primers used in this study were listed in Table S1.

### 2.4. Plasmid Construction and Transformation

The coding sequence and the promoter of *PtoWUSa* were amplified and cloned into pDNOR222.1 for sequencing. The right coding sequence was sub-cloned respectively into pMDC32 and pMDC7, represented as p*35S*::*PtoWUSa* and p*XVE*::*PtoWUSa*. The right promoter sequence was conducted into pMDC164, represented as p*PtoWUSa*::*GUS*. The resulting constructs were then transformed into *Agrobacterium GV3101* by electroporation. Agrobacterium carrying p*PtoWUSa*::*GUS*, p*35S*::*PtoWUSa*, or p*XVE*::*PtoWUSa* were used for poplar transformation. The RNAs were extracted from roots of two-week-old p*35S*::*PtoWUSa* transgenic *A. thaliana* plants and three-week-old p*35S*::*PtoWUSa* transgenic poplar plants, and the transcription levels of *PtoWUSa* were detected using qRT-PCR. Two independent transgenic *A. thaliana* lines (#31 and #43) and two independent transgenic poplar lines (#16 and #18) with high *PtoWUSa* abundance were used for further study. The p*PtoWUSa*::*GUS* was used for GUS staining according to the previous studies [39].

### 2.5. Estradiol Treatments

The 17 β-estradiol was dissolved in dimethyl sulfoxide (DMSO) as 10 mM stock solution. The apicals (2–3 cm) from 84 K and two independent p*XVE*::*PtoWUSa* transgenic lines (#26 and #27) with two leaves were induced by adding 17 β-estradiol into the nutrient solution, while the untreated condition was corresponded to plants treated with DMSO. Phenotypic observation was carried out on 20-day-old poplars. The concentration of 17 β-estradiol was determined based on treatment in poplar [40].

### 2.6. Statistical Analysis

The statistical analysis of all the experimental data was performed with SPSS 16.0 software (SPSS Inc, Chicago, USA). Significant differences were detected using *t* test and the differences were considered significant if *p* < 0.05.

## 3. Results

### 3.1. Phylogenetic Analysis and Protein Sequence Comparisons

**Figure 1:**
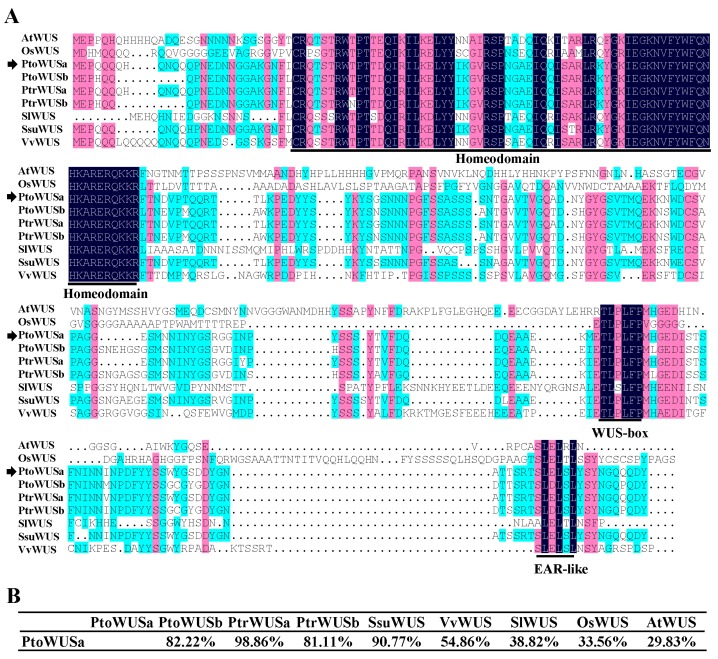
PtoWUSa was highly conserved in a broad range of plant species. (**A**) Amino acid sequence alignment of PtoWUSa with AtWUS (*Arabidopsis thaliana*), OsWUS (*Oryza sativa*), PtrWUS (*Populus trichocarpa*), SlWUS (*Solanum lycopersicum*), SsuWUS (*Salix suchowensis*), and VvWUS (*Vitis vinifera*) orthologous genes in other plant species. The conserved regions (homeodomain, WUS-box motif, and ethylene-responsive element binding factor associated amphiphilic repression (EAR)-like domain) were underlined. (**B**) WUS amino acid sequence identity.

Two *P. tomentosa WOXs* (*PtoWUSa* and *PtoWUSb*) were identified as the orthologous genes of *AtWUS* in our previous study, but only *PtoWUSa* was expressed in AR [37]. Therefore, *PtoWUSa* was chosen for function investigation in AR development in the present study. PtoWUSa contained a helix-loop-helix-turn domain in the N-terminal which was known as the homeodomain (HD), an EAR-like domain in the C-terminal, and a conserved WUS-box domain (TLxLFP) between them (Figure 1). The result of phylogenetic relationships between PtoWUSa and AtWOXs showed that PtoWUSa belongs to the WUS/modern clade and had a close genetic relationship with AtWUS (Figure S1). Compared with WUS protein sequences from different species, PtoWUSa shared 29.83–98.86% identity with WUS sequences from other species (Figure 1). PtoWUSa and PtrWUSa shared 98.86% identity and had only three residues difference over the entire amino acid sequence. These results indicate that WUS had conserved domain, although the sequences were variable in different plant species.

### 3.2. Expression Pattern of PtoWUSa

**Figure 2:**
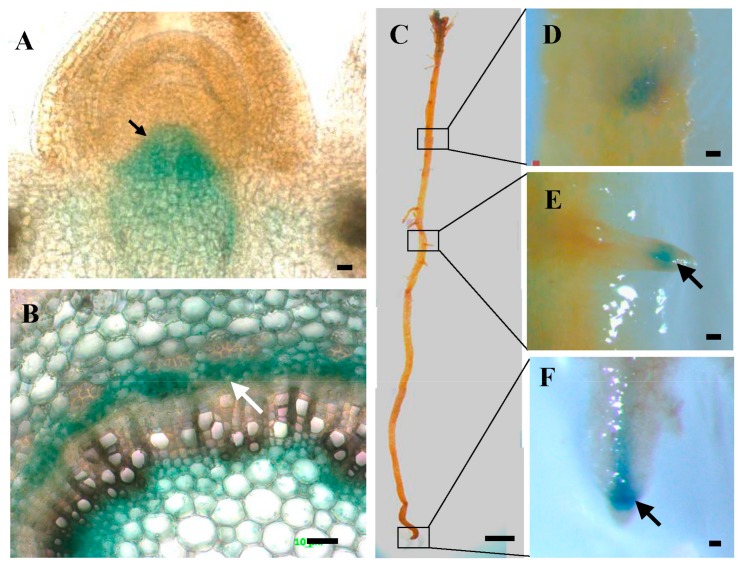
Spatial expression patterns of *PtoWUSa* detected by GUS staining. (**A**,**B**) Spatial expression patterns of *PtoWUSa* in the longitudinal section of shoot apex (**A**) and cross-section of stem (**B**). (**C**–**F**) Expression patterns of *PtoWUSa* in root (**C**); LRT (**D** and **E**) and ART (**F**) were close-ups of the boxed regions in (**C**). Arrows highlighted the GUS signal. Scale bars: (**A**) = 20 μm; (**B**) = 10 μm; (**C**) = 5 mm; (**D**,**E**) = 500 μm.

The GUS reporter of *PtoWUSa* was conducted to confirm the expression pattern of *PtoWUSa*. GUS staining, showing that the GUS signals were detected in the SAM (Figure 2A) and stem (Figure 2B) and roots (Figure 2C–F). The roots from the poplar stem were named ARs, and the roots located in the ARs were named LRs. In roots, the GUS signal was specially detected in the AR tip (ART) and the LR tip (LRT) (Figure 2C–F). These results indicated that the *PtoWUSa* accumulated in LRT and ART in the roots, suggesting that *PtoWUSa* might play roles in root development.

### 3.3. Ectopic Expression PtoWUSa Affected Root Development in Arabidopsis

**Figure 3:**
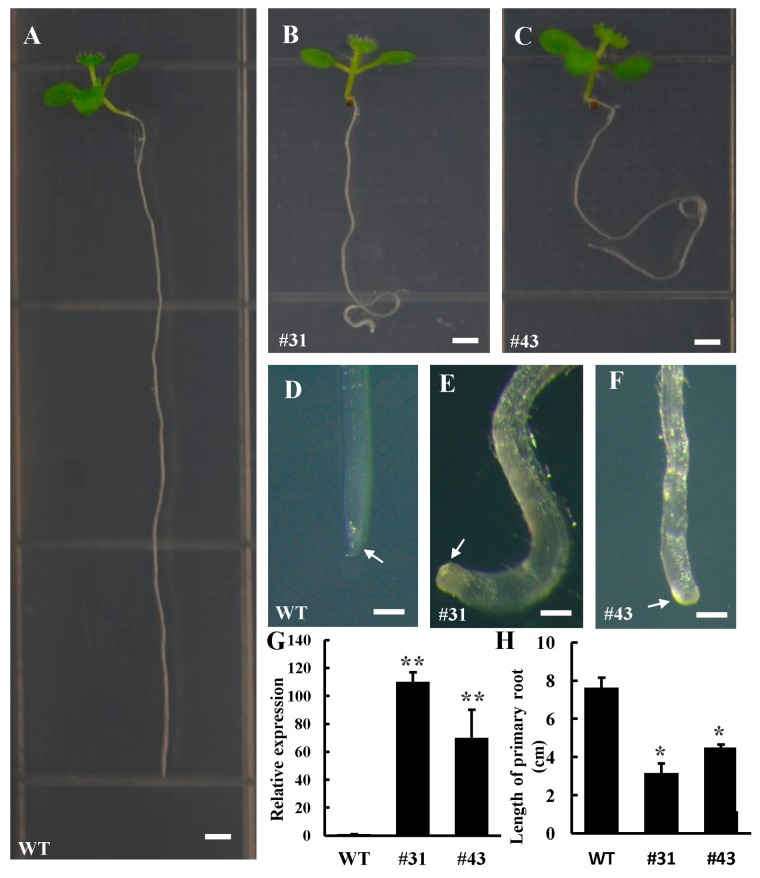
Reduced root length and changed morphology by overexpression of *PtoWUSa* in *Arabidopsis*. (**A**–**C**) two-week-old of WT (**A**) and *PtoWUSa*-overexpressing *Arabidopsis* plants including two independent transgenic lines of #31 (**B**) and #43 (**C**). (**D**–**F**) The primary root of WT (**D**) and two transgenic lines (**E**,**F**). Arrows pointed to primary root tip. (**G**) Transcription levels of *PtoWUSa* in transgenic plants. *AtACTIN* was used as a reference gene. The expression level in WT was set to 1. (**H**) The length of root of WT and transgenic plants. * and ** indicated the difference between WT and each transgenic line at *p* < 0.05 and *p* < 0.01 levels, respectively. Scale bars: (**A–C**) = 0.2 cm; (**D**–**F**) = 200 μm.

**Figure 4:**
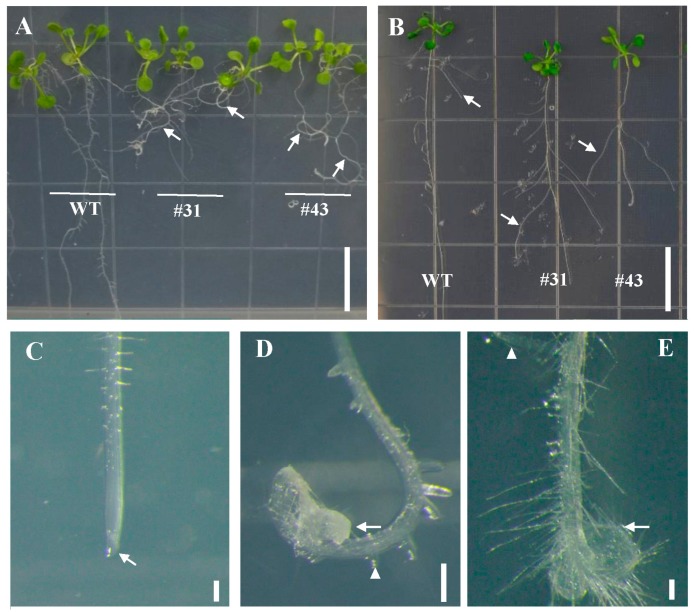
Disordered root morphology and enhanced lateral root length by overexpression of *PtoWUSa* in *Arabidopsis*. (**A**) Three-week-old WT and *PtoWUSa*-overexpressing *Arabidopsis* plants. (**B**) Comparison of straightening lateral roots (LRs). (**C**–**E**) The primary root of WT and transgenic plants. Arrows pointed to primary root tip; triangles pointed to LR. Scale bars: (**A**,**B**) =1.5 cm; (**C**–**E**) =100 μm.

As the herb model plant, *Arabidopsis* has a rapid lifecycle and easy transformation system. Therefore, we firstly investigated the function of *PtoWUSa* by ectopic expression of *PtoWUSa* in *Arabidopsis.* Two independent transgenic lines (#31 and #43) with high abundance of *PtoWUSa* (Figure 3G) were chosen for functional study. After cultured on 1/2 MS medium for two weeks, the length of primary roots of *PtoWUSa*-overexpressing *Arabidopsis* seedlings was severely inhibited and was only 50% of WT seedlings (Figure 3A–C,H). Interestingly, the roots of transgenic plants appeared to curve and showed agravitropism (Figure 3A–C), and their root tip was enlarged and swollen (Figure 3D–F). After three weeks of growth, the abnormal root morphology of transgenic plants was intensified, such as the decreased primary root length, agravitropism, and curved root (Figure 4). The root hair and LRs were located closer to the root tip in transgenic plants, being different with WT plants (Figure 4F,H). It is noteworthy that the LR length of transgenic plants was increased compared with WT plants when the transgenic LRs were stretched out (Figure 4B).

**Figure 5:**
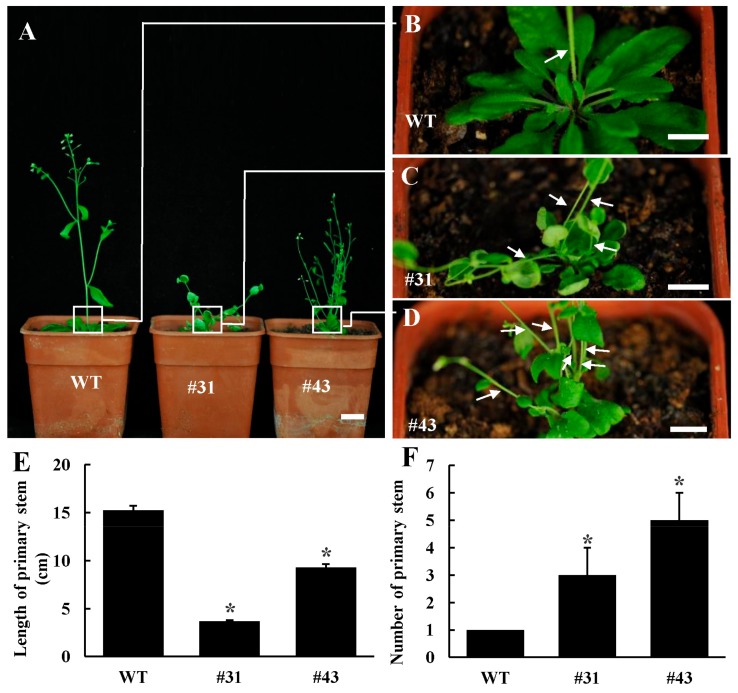
Overexpression of *PtoWUSa* led to compromise of shoot branching in *Arabidopsis*. (**A**) Aboveground phenotypes of WT and *PtoWUSa*-overexpressing *Arabidopsis* plants. (**B**–**D**) Close-up views of shoot branches in the squares of the corresponding plants in (**A**). Arrows pointed to shoot branches. (**E**–**F**) Comparison of length of primary stem (**E**) and number of primary stem (**F**) between WT and transgenic plants. * indicated the difference between Ctrl and each transgenic line at *p* < 0.05 level, respectively. Scale bars: (**A**) = 2 cm; (**B**–**D**) = 1 cm.

Moreover, the *PtoWUSa*-overexpressing *Arabidopsis* plants exhibited more branches than WT plants (Figure 5A–D), and the stem number of transgenic plants reached to four to six (Figure 5F), but the height of primary stem of transgenic plants was reduced to 1/3–1/2 of WT plants after growing on soil for four weeks (Figure 5E).

### 3.4. Overexpression of PtoWUSa also Alters the Morphology of ARs in Poplar

**Figure 6:**
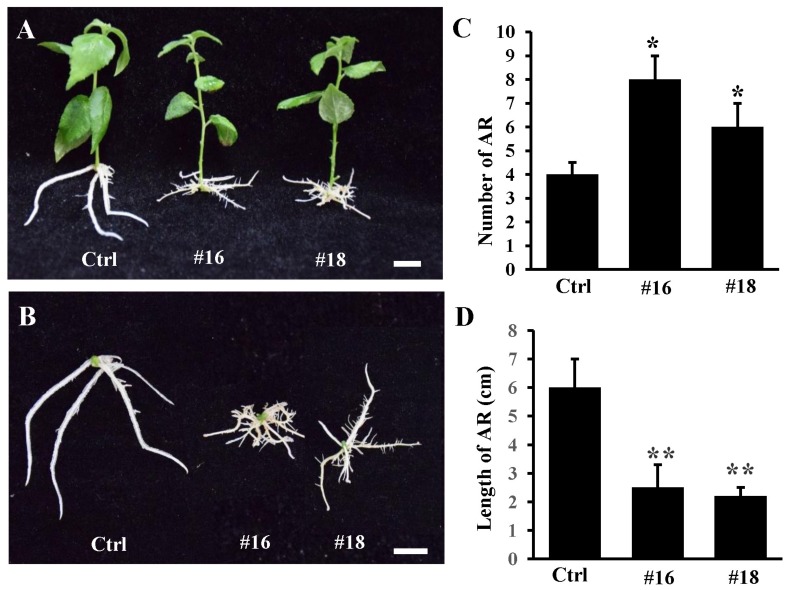
Overexpression of *PtoWUSa* promoted AR formation and inhibited its elongation in poplar. (**A**,**B**) Comparison of three-week-old Ctrl and *PtoWUSa*-overexpressing poplar plants including two independent transgenic lines #16 and #18. (**C**,**D**) Comparison of AR number (**C**) and AR length (**D**) between Ctrl and transgenic plants. * and ** indicated the difference between Ctrl and each transgenic line at *p* < 0.05 and *p* < 0.01 levels, respectively. Scale bar: 1 cm.

**Figure 7:**
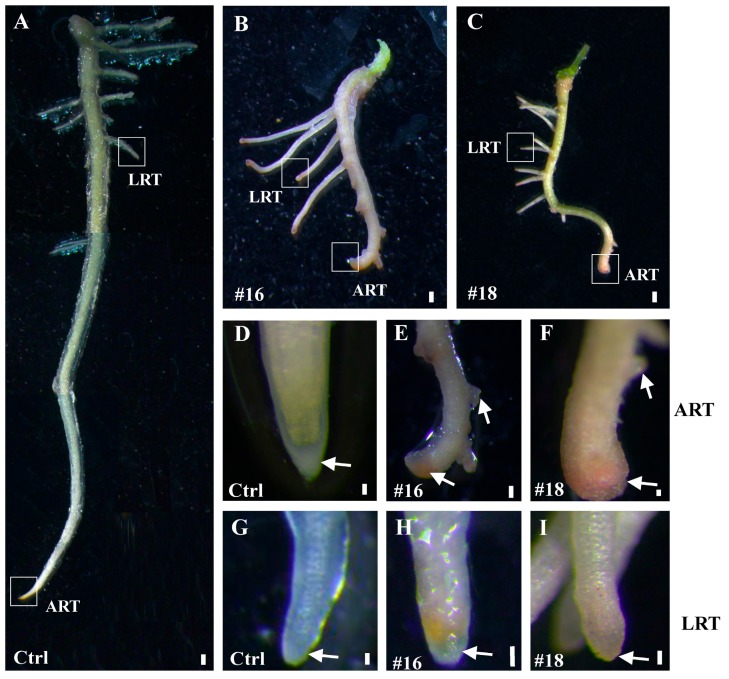
Root morphology of Ctrl and *PtoWUSa-*overexpressing poplar plants. (**A**–**C**) ARs of Ctrl and transgenic plants. (**D**,**F**) Close-up views of ARTs of Ctrl and transgenic plants from (**A**–**C**). (**G**–**I**) Close-up views of LRTs of Ctrl and transgenic plants from (**A–C**). Scale bars: (**A**–**C**) = 1 mm; (**D**,**F**–**I**) = 1 μm; (**E**) = 3 μm.

In order to investigate whether *PtoWUSa* performed similar functions in poplar, we generated transgenic poplar plants overexpressing *PtoWUSa*. Two independent transgenic lines (#16 and #18) with high abundance of *PtoWUSa* were used for further study. After cultured for three weeks, the number of ARs increased to 6 to 8 in transgenic seedlings, which was about twice in control (Ctrl) (Figure 6A–C). Additionally, the AR length of *PtoWUSa*-overexpressing poplar plants (~2 cm) was significantly less than that of Ctrl plants (~6 cm) (Figure 6A–C). Moreover, the morphology of ARTs and LRTs in Ctrl plants was always sharp and small, whereas ARTs and LRTs became enlarged and swollen in transgenic plants. The location of LRs was closer to the ARTs in transgenic plants than in Ctrl plants (Figure 7).

**Figure 8:**
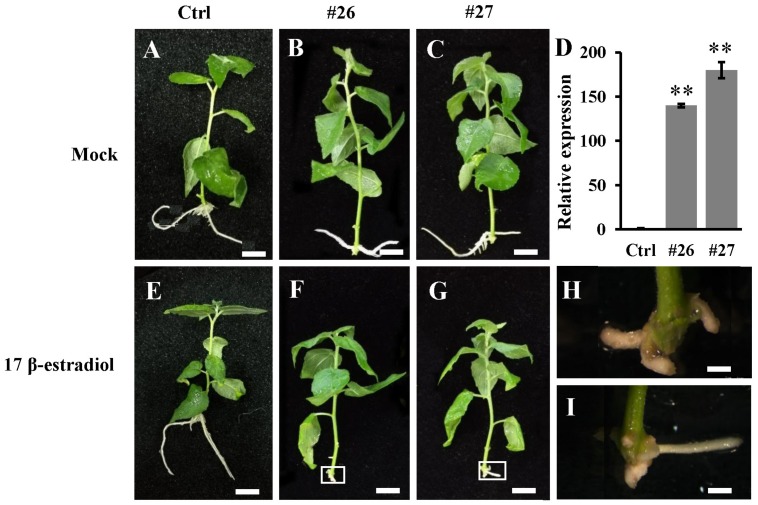
Growth phenotype of Ctrl and p*XVE*::*PtoWUSa* transgenic plants (including two independent transgenic lines #26 and #27) treated with 0 μM (**A–C**) and 10 μM (**E**–**G**) 17 β-estradiol for three weeks. (**D**) Expression levels of *PtoWUSa* in Ctrl and transgenic plants after treated 10 μM 17 β-estradiol. (**H**,**I**) Close-up views of ARs of transgenic line #26 from (**F**) and #27 from (**G**). *PtoACTIN* was used as reference gene. The expression level in Ctrl was set to 1. ** indicated the difference between Ctrl and each transgenic line at *p* < 0.01 level. Scale bar: (**A–C**,**E–G**) = 1 cm; (**H**,**I**) = 3 cm.

To further confirm the functions of the *PtoWUSa*, we constructed an inducible expression vector p*XVE*::*PtoWUSa* and transformed it into poplar. After being induced under 10 μM 17 β-estradiol for 15 days, the two transgenic lines (#26 and #27) with high expression of *PtoWUSa* were chose for subsequence study (Figure 8D). There was no different phenotype between the Ctrl and transgenic plants without 17 β-estradiol treatment (Figure 8A–C). After treatment by 17 β-estradiol, the AR primordia was observed, AR elongation was inhibited and the ARTs became enlarged and swollen in the p*XVE*::*PtoWUSa* transgenic plants (Figure 8E–I). All these results indicated that *PtoWUSa* was involved in AR development in poplar.

### 3.5. Expression Analysis of Auxin-Related Genes in PtoWUSa-Overexpressing Transgenic Poplar

**Figure 9:**
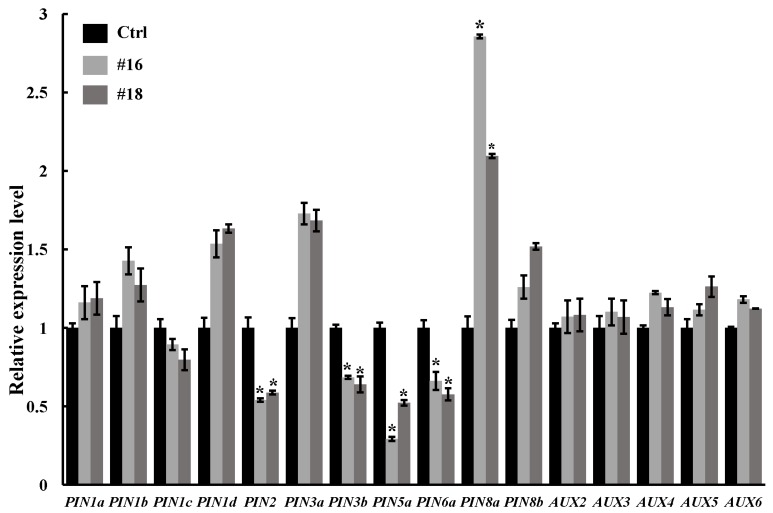
Expression analysis of auxin transport genes. Expression levels of *PINs* and *AUX1*-*like* genes in three-week-old Ctrl and *PtoWUSa*-overexpressing poplar plants were detected using qRT-PCR. The expression levels of each gene in Ctrl were set to 1. * indicated the difference between Ctrl and each transgenic line at *p* < 0.05 level.

The extensive researches have been shown that auxin acts as an important regulator in root development. The root morphology of transgenic plants overexpressing *PtoWUSa* was similar to the *pin* or *aux* mutants. This observation impelled us to clarify whether this phenotype in the present study was caused through altered distribution of auxin in transgenic plants. Thus, we examined the expression of auxin transport genes including *AUX* and *PIN* in the ARs of three-week-old *PtoWUSa*-overexpressing plants and Ctrl plants. The expression levels of eleven *PINs* and five *AUXs* were successfully detected. Among *PIN* genes, the expression levels of five *PIN* genes (*PIN1c*, *PIN2*, *PIN3b*, *PIN5a,* and *PIN6a*) for auxin polar transport were reduced, while other genes were slightly up-regulated (Figure 9). The expression levels of *AUXs* were no obvious change (Figure 9). These results suggested that overexpression of *PtoWUSa* might affect polar transport in the ARs by regulating the expression of *PINs*.

## 4. Discussion

*WUS* is the founding member of the *WOX* family, which is the plant-specific transcription factor. It has been well documented that *WUS* is expressed in SAM and FM, and its main function is to maintain stem cell balance and to keep stem cells balance between division and differentiation in herbage plant. However, research on the homologues of *WUS* in woody plant is lacking and its function is unclear. In this study, the function of poplar *PtoWUSa* was characterized. The result of sequence analysis showed that PtoWUSa was a member of the WUS clade containing the conserved domains of HD, WUS-box, and EAR-like domain. Expression patterns exhibited that *PtoWUSa* was not only expressed in SAM, but was also expressed in root and stem, which was different from expression patterns of other *WUS* that have been reported. It has been confirmed that *WUS* also plays role in its expressed zone, such as flower and shoot meristem in *Arabidopsis* [26,27]. Therefore, its expression patterns indicated that *PtoWUSa* might participate in root and stem development.

In our study, multiple stems were present in *PtoWUSa*-overexpressing plants, implying that *PtoWUSa* affected the meristem growth. Moreover, the root development was disordered in *Arabidopsis* and poplar, indicating that *PtoWUSa* was involved in AR development. It is reported that *WUS* play key role in shoot regeneration. The *atwus* mutant is defective in shoot meristem development, and overexpression of *AtWUS* can promote the transition from vegetative stage to embryonic stage, and *GhWUS* is also involved in the formation of the embryogenic callus in cotton [36]. However, being different in *AtWUS* and *GhWUS* transgenic plants [36,41,42], embryo-like structures were not detected in the root tip of *PtoWUSa*-overexpressing *Arabidopsis* and poplar plants. These results implied that *PtoWUSa* might have a weaker function in somatic embryogenic than *AtWUS* and *GhWUS*.

*WOX* transcription factors have been found to be involved in AR development. In *Arabidopsis*, *AtWOX7* from WUS-clade is involved in LR development and it is activated by *AtWOX11*/*12* in the process of AR de novo organogenesis [25,37]. However, no *AtWOX7* homolog gene was identified in poplar. Based on the expression pattern and evolutionary relationship, we speculated that *PtoWUSa*, *PtoWOX5a* or other *PtoWOXs* might take place of the roles of *WOX7* in poplar AR development. In poplar, a member of WUS clade, *PtoWOX5a* also expressed in the ARs, and an overexpression of *PtoWOX5a* affected the morphology of ARs and LRs [39]. *WOX5* can rescue the deficiencies of *wus* mutants in *Arabidopsis*, indicating that *WUS* and *WOX5* has similar function [43]. Furthermore, the similar phenotype of ARs in *PtoWUSa*- and *PtoWOX5a*-overexpressing plants indicated that *PtoWUSa* and *PtoWOX5a* also had similar function in AR development in poplar. The direct genetic evidences including CRISR-cas9 or RNAi need to further reveal its function in AR development in future.

In *A*. *thaliana*, *WUS* has been demonstrated to be involved in meristem growth and maintenance via acting its down-stream genes, such as *CLV3* and *type-A Arabidopsis Response Regulator 5/7/15* (*ARR5/7/15*). In SAM, WUS can directly activate *CLV3* and in turn the *CLV3* signal represses *WUS* expression. This *WUS*-*CLV* feedback loop controls SAM maintenance [28,29]. *WUS* is linked to cytokinin signaling through direct transcriptional repression of *ARR5/7*/*15*, which are negative regulators of cytokinin signaling and are necessary for proper meristem function [44]. In our study, we also found that overexpression of *PtoWUSa* repressed the expression of five *PIN* genes (*PIN1c*, *PIN2*, *PIN3b*, *PIN5a* and *PIN6a*) in ARs in poplar. PIN auxin efflux carriers and AUX1-like auxin influx carriers are two classes of proteins for polar auxin transport [10]. The *PIN* function has been confirmed to take part in polar auxin transport, which is important for root morphogenesis [2,45]. *OsPIN1* is expressed in root primordial, and suppressing the expression of *OsPIN1* significantly inhibits ARs development through affecting auxin transport [8,46]. *AtPIN2* plays an important role in controlling gravitropism by regulating the redistribution of auxin from the stele towards the elongation zone of roots. In *pin2* mutant, the root showed agravitropism and reduced elongation [7]. In our previous study, it has been confirmed that *PtPIN1*, *PtPIN2,* and *PtPIN5*a are specifically expressed in ARs, implying that they are involved in AR development by regulating the polar auxin transport in ARs [47]. In *PtoWUSa*-overexpressing plants, the expression levels of five *PtPIN*s (*PIN1c*, *PIN2*, *PIN3b*, *PIN5a* and *PIN6a*) were down-regulated, and this might influence polar auxin concentration and result in agravitropism of transgenic plant (Figure 4 and Figure 5). In the *pin2* and *pin5* mutants, the root elongation was inhibited [7,48]. The reduced expression of *PtPIN5a* and *PtPIN2* might be the reason why the AR elongation was inhibited in *PtoWUSa*-overexpressing plant in our study (Figure 6). No obvious change of expression levels of *AUX1-like* suggested that *PtoWUSa* was mainly regulated the efflux transport of auxin, rather than auxin influx carrier. These studies indicate that *PtoWUSa* may have superimposed coordination of reprogramming towards developmental plasticity, and it processes multiple regulatory mechanisms and biological functions. Further investigations need to be carried out to reveal its complex regulatory network in future.

## 5. Conclusions

In this study, we explored the function of *PtoWUSa* and found it is involved in AR development by down-regulating the expression of auxin efflux carrier in *PtoWUSa*-overexpressing plants. Constitutive expression of *PtoWUSa* caused the defect of root development, including agravitropism, and enlarged and swollen root tip in transgenic *Arabidopsis* and poplar plants. Our results provide some new information about function of *WOX* in poplar, and this study will help us gain a better understanding the AR development in woody plant.

## Supplementary Materials

The following are available online at https://www.mdpi.com/2073-4425/11/2/176/s1. Figure S1. Phylogenetic analysis of PtoWUSa and AtWOXs. The 18 proteins were clustered into three distinct groups: the modern/WUS clade, an ancient clade, and an intermediate clade. Table S1. The primers sequences used in this study.
