# Supplementary figures and images for: The WUSCHELa (PtoWUSa) is Involved in Developmental Plasticity of Adventitious Root in Poplar
Source: Genes (Basel). 2020 Feb 6;11(2):176. doi: 10.3390/genes11020176 (PMC7073988; doi:10.3390/genes11020176)

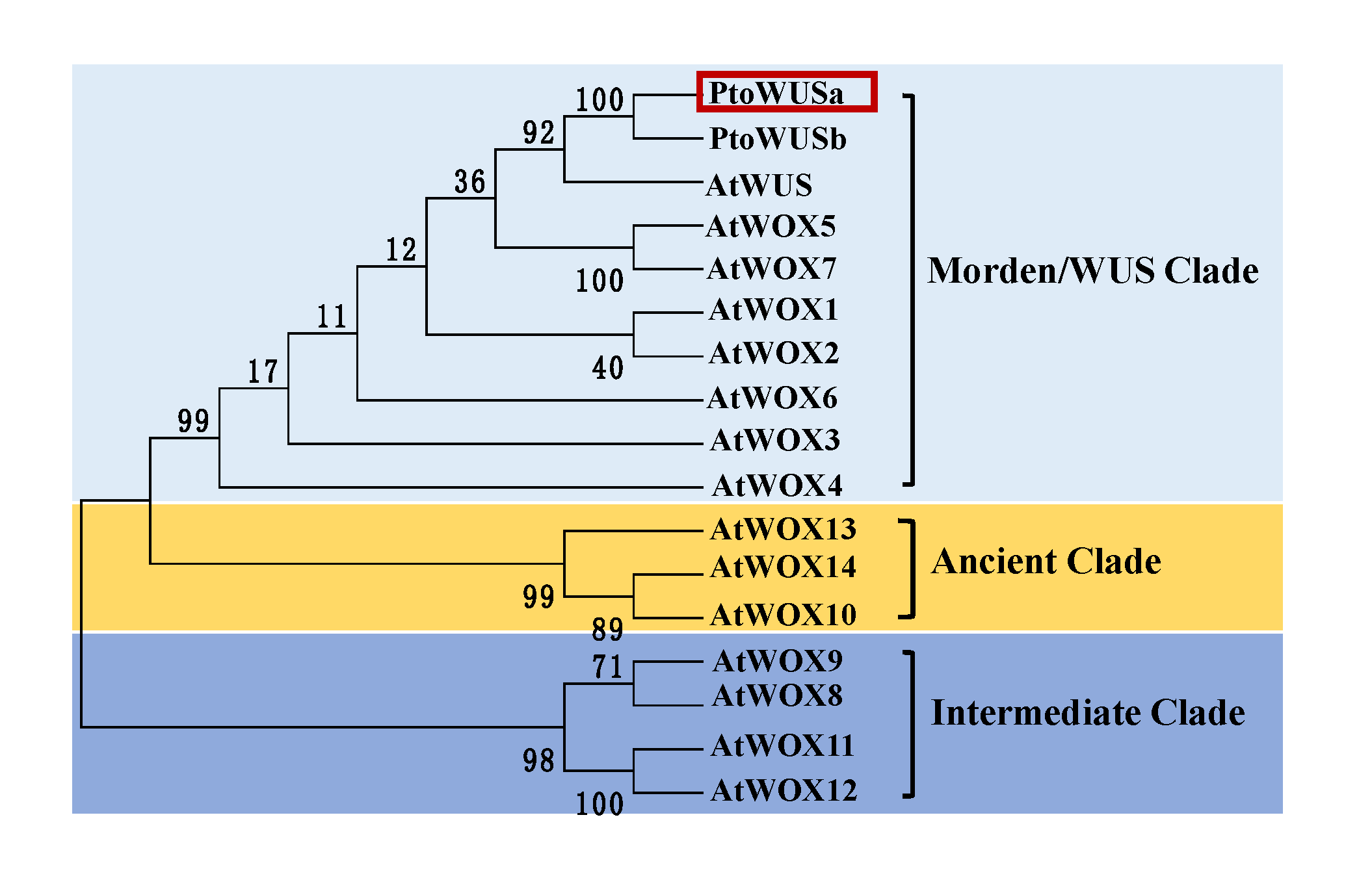

Supplement: Supplementary file 1 [file genes-11-00176-s001.zip › Supplementary File/Fig S1.tif]
